# Identification of a prognostic ferroptosis-related lncRNA signature in the tumor microenvironment of lung adenocarcinoma

**DOI:** 10.1038/s41420-021-00576-z

**Published:** 2021-07-26

**Authors:** Yugang Guo, Zhongyu Qu, Dandan Li, Fanghui Bai, Juan Xing, Qian Ding, Jiawei Zhou, Lunguang Yao, Qian Xu

**Affiliations:** 1 Henan Provincial Engineering Laboratory of Insects Bioreactor, Nanyang Normal University, Nanyang, China; 2 Henan Provincial Nanyang Central Hospital, Nanyang, China; 3 School of Chemistry and Pharmaceutical Engineering, Nanyang Normal University, Nanyang, China

**Keywords:** Immunosuppression, Prognostic markers, Non-small-cell lung cancer

## Abstract

Ferroptosis is closely linked to various cancers, including lung adenocarcinoma (LUAD); however, the factors involved in the regulation of ferroptosis-related genes are not well established. In this study, we identified and characterized ferroptosis-related long noncoding RNAs (lncRNAs) in LUAD. In particular, a coexpression network of ferroptosis-related mRNAs and lncRNAs from The Cancer Genome Atlas (TCGA) was constructed. Univariate and multivariate Cox proportional hazards analyses were performed to establish a prognostic ferroptosis-related lncRNA signature (FerRLSig). We obtained a prognostic risk model consisting of 10 ferroptosis-related lncRNAs: AL606489.1, AC106047.1, LINC02081, AC090559.1, AC026355.1, FAM83A-AS1, AL034397.3, AC092171.5, AC010980.2, and AC123595.1. High risk scores according to the FerRLSig were significantly associated with poor overall survival (hazard ratio (HR) = 1.412, 95% CI = 1.271–1.568; *P* < 0.001). Receiver operating characteristic (ROC) curves and a principal component analysis further supported the accuracy of the model. Next, a prognostic nomogram combining FerRLSig with clinical features was established and showed favorable predictive efficacy for survival risk stratification. In addition, gene set enrichment analysis (GSEA) revealed that FerRLSig is involved in many malignancy-associated immunoregulatory pathways. Based on the risk model, we found that the immune status and response to immunotherapy, chemotherapy, and targeted therapy differed significantly between the high-risk and low-risk groups. These results offer novel insights into the pathogenesis of LUAD, including the contribution of ferroptosis-related lncRNAs, and reveal a prognostic indicator with the potential to inform immunological research and treatment.

## Introduction

Lung cancer is one of the leading causes of cancer-related deaths worldwide [1, 2]. Although integrative therapies such as molecular targeted therapy, chemotherapy, and radiotherapy have been developed for lung adenocarcinoma (LUAD), the 5-year overall survival (OS) rate is only 15% [3, 4]. Thus, it is essential to identify effective diagnostic markers, therapeutic targets, and prognostic factors.

Ferroptosis is a newly identified type of programmed cell death that is distinguished from apoptosis, necrosis, pyroptosis, and autophagic cell death [5, 6]. It induces cell injury or death via the iron-dependent lipid peroxidation process [7–9]. Ferroptosis-related genes have been identified as diagnostic markers and could even be potential drug targets, especially for the development and pharmacological study of anticancer agents and cancer therapies [10]. Ferroptosis plays an important role in the development of lung cancer [11–15]. However, its precise roles in the origin and progression of LUAD remain unclear.

Furthermore, the role of ferroptosis in the tumor immune microenvironment is not well established. Recent studies have demonstrated that immunotherapy-activated CD8+ T cells enhance ferroptosis-specific lipid peroxidation in tumor cells and increase the efficacy of cancer immunotherapy [16, 17]. Intriguingly, IFNγ released from CD8+ T cells downregulates the expression of SLC7A11 and SLC3A2, thereby limiting cystine uptake by tumor cells and promoting tumor cell lipid peroxidation and ferroptosis [17]. This indicates that IFNγ is a vital mediator of tumor immune evasion and a potential target for improving clinical responses to immunotherapy. Furthermore, Lai et al. [11] identified a relationship between ferroptosis and non-small cell lung carcinoma prognosis. However, a comprehensive understanding of the mechanisms underlying the effects of ferroptosis-related genes in LUAD and their functions is lacking.

Long noncoding RNAs (lncRNAs), which are >200 nucleotides in length and have limited protein coding ability, play essential roles in the development and progression of diverse cancers, including LUAD. The dysregulation of lncRNAs has been implicated in tumor cell proliferation, invasion, metastasis, and chemoresistance in lung cancer [18–21]. In addition, lncRNAs have been a major focus of research into ferroptosis [20, 22, 23]. For example, the p53-related lncRNA P53RRA is involved in the regulation of SLC7A11, which is critical for the sensitivity of ferroptotic responses [23].

In the present study, we constructed a ferroptosis-related lncRNA signature and systematically assessed the correlations of ferroptosis-related lncRNAs with the prognosis and clinicopathological features of LUAD patients. We then established a nomogram that incorporates the ferroptosis-related lncRNA signature (FerRLSig) and clinical factors to predict the survival of these patients. The high-risk and low-risk groups were compared with respect to various factors, including immune status and response to immunotherapy. Our results provide valuable insights into the regulatory mechanisms by which ferroptosis contributes to LUAD and may help to improve the effectiveness of individualized treatment and assessments of prognosis.

## Results

### Identification of ferroptosis-related lncRNAs with prognostic value in LUAD

**Fig. 1:**
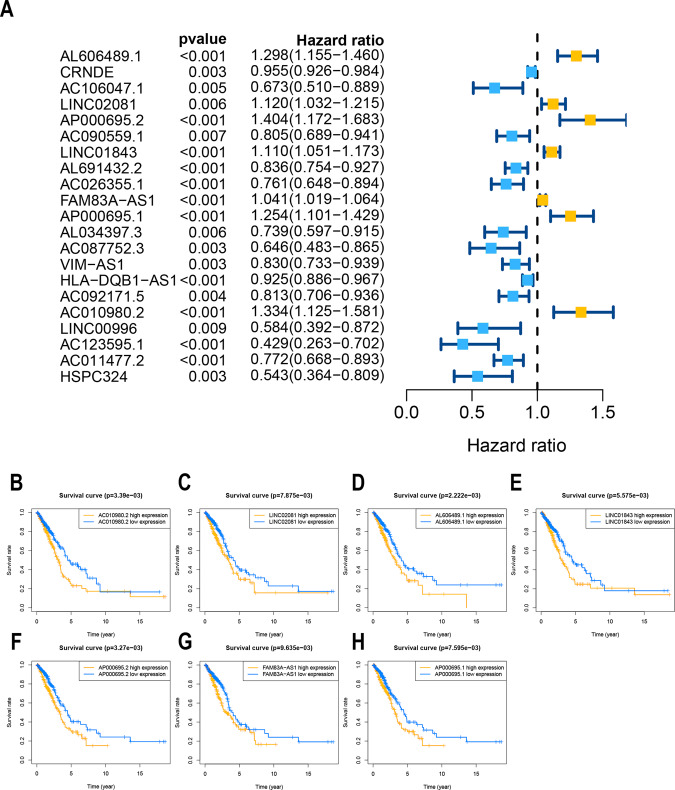
Identification of ferroptosis-related lncRNAs with significant prognostic value in LUAD. **A** Forest plot showing the HR (95% CI) and *P* values for selected lncRNAs determined using univariate Cox proportional hazards analysis (all *P* < 0.01). Fourteen lncRNAs were associated with a good prognosis, and 7 lncRNAs were associated with a poor prognosis. Kaplan–Meier survival curves for seven unfavorable prognostic ferroptosis-related lncRNA markers: (**B**) AC010980.2, (**C**) LINC02081, (**D**) AL606489.1, (**E**) LINC01843, (**F**) AP000695.2, (**G**) FAM83A-AS1, and (**H**) AP000695.1 (*P* < 0.001).

We first screened 174 ferroptosis-related genes (mRNAs), and expression data were available for 169 of these genes in TCGA LUAD (Table S1). Then, 1621 ferroptosis-related lncRNAs were identified by Pearson correlation analysis (|*R*^2^ | > 0.3, *P* < 0.001). Subsequently, 21 lncRNAs whose expression levels were correlated with patient outcomes, suggesting that they had prognostic value for LUAD, were screened by univariate Cox regression (*P* < 0.01, Fig. 1A, Table S2). Seven lncRNAs were identified as poor prognostic factors (HR > 1, Fig. 1B), and 14 were identified as favorable prognostic factors.

### Identification of a prognostic ferroptosis-related lncRNA signature

**Fig. 2:**
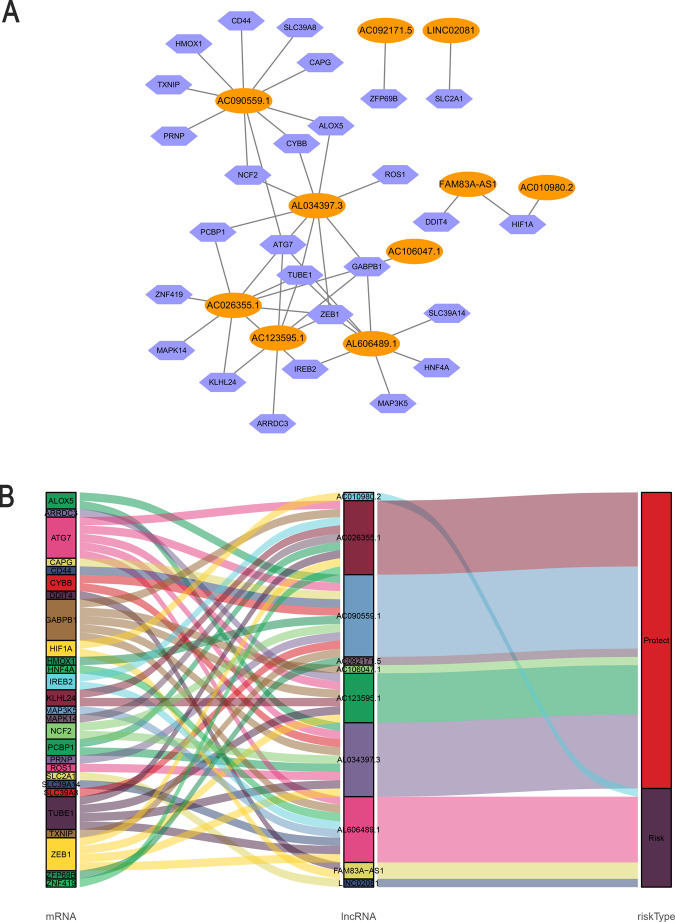
Coexpression network and Sankey diagram of prognostic ferroptosis-related lncRNAs. **A** A coexpression network of ferroptosis-related lncRNAs and mRNAs was constructed and visualized using Cytoscape. Orange ellipses indicate prognostic lncRNAs, and blue violet diamonds indicate ferroptosis-related mRNAs. The levels of the 10 ferroptosis-related lncRNAs were associated with the levels of 27 ferroptosis mRNAs. **B** Sankey diagram showing the associations between prognostic ferroptosis-related lncRNAs, mRNAs, and risk type.

**Fig. 3:**
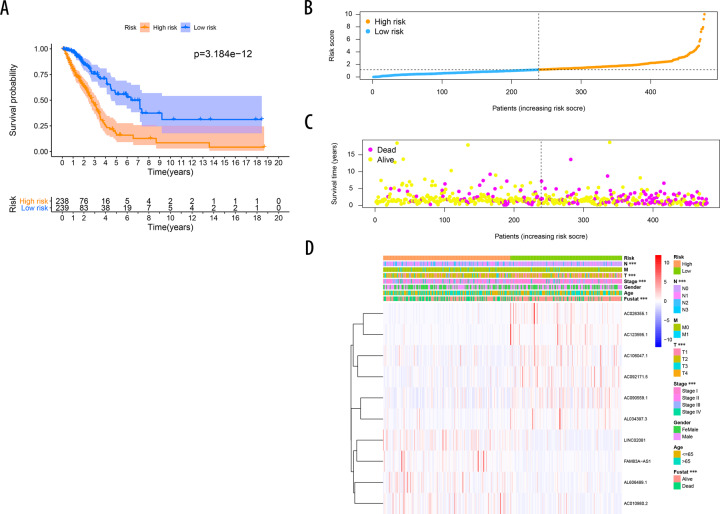
Prognostic value of the risk model including 10 ferroptosis-related lncRNAs. **A** Kaplan–Meier curves for OS in the high-risk and low-risk groups stratified by FerRLSig (*P* < 0.0001). **B** Risk curve based on the risk score for each sample, where orange indicates a high risk and blue indicates a low risk. **C** Scatterplot based on the survival status of each sample. Yellow and violet dots indicate survival and death, respectively. **D** The heatmap shows the distribution of 10 ferroptosis-related lncRNAs and clinicopathological parameters in the high-risk and low-risk groups in the TCGA-LUAD cohort.

Ten ferroptosis‐related lncRNAs were identified in a multivariate Cox proportional hazards regression analysis (AL606489.1, AC106047.1, LINC02081, AC090559.1, AC026355.1, FAM83A-AS1, AL034397.3, AC092171.5, AC010980.2, and AC123595.1) and were used to establish a prognostic ferroptosis-related lncRNA signature (FerRLSig). Correlations between the levels of these 10 lncRNAs and ferroptosis genes are shown in Fig. 2A. Among these, four lncRNAs (AL606489.1, LINC02081, FAM83A-AS1, and AC010980.2) were significant independent unfavorable prognostic factors, while the remaining lncRNAs (AC106047.1, AC090559.1, AC026355.1, AL034397.3, AC092171.5, and AC123595.1) were independent favorable prognostic factors for OS (Fig. 2B). The risk score formula was as follows: 0.1567 × AL606489.1 + (−0.2795 × AC106047.1) + (0.1157 × LINC02081) + (−0.2422 × A). (C090559.1) + (−0.24 × AC026355.1) + (0.0207 × FAM83A-AS1) + (−0.1425 × AL034397.3) + (−0.1195 × AC092171.5) + (0.3016 × AC010980.2) + (−0.4327 × AC123595.1). We calculated the risk score for each patient based on personalized FerRLSig levels. Using the median risk score as the threshold value, we divided patients into low‐risk (*n* = 238) and high-risk groups (*n* = 239). Kaplan–Meier survival curves showed a significant difference in OS between the high-FerRLSig and low-FerRLSig groups for patients with LUAD (*P* < 0.0001, Fig. 3A), suggesting that the newly developed signature effectively predicts survival. The distribution of the expression of 10 lncRNAs, clinicopathological factors, survival status, and risk scores are shown in Fig. 3B–D.

### Evaluation of FerRLSig as an independent prognostic factor for LUAD

**Fig. 4:**
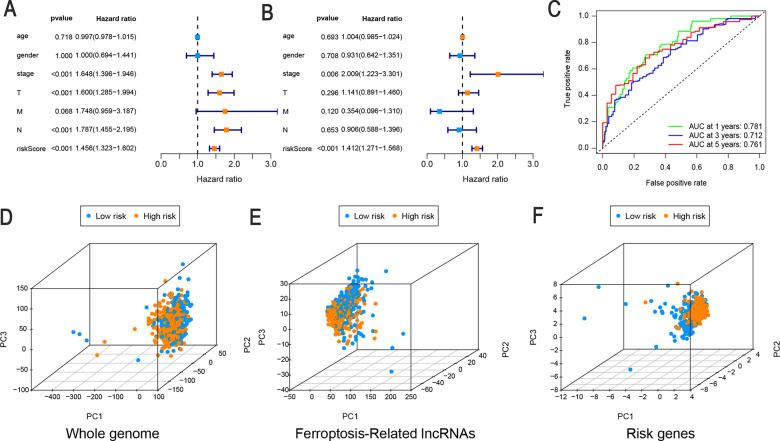
FerRLSig is an independent prognostic factor for overall survival. Univariate (**A**) and multivariate (**B**) Cox regression analyses of associations between clinical parameters (including FerRLSig) and OS. **C** Time-dependent ROC curves of OS at 1, 3, and 5 years. Principal component analysis (PCA) of low-risk and high-risk groups based on the (**D**) whole-genome, (**E**) ferroptosis-related lncRNAs, and (**F**) the risk model including 10 ferroptosis-related lncRNAs; patients with high risk scores are indicated in orange, and those with high risk scores are indicated in blue. T, tumor stage; N stage, lymph node metastasis stage; M stage, distant metastasis stage.

Next, univariate and multivariate Cox regression analyses were performed to determine whether FerRLSig is an independent prognostic model for OS in patients with LUAD. The HRs (95% CI) for the risk score were 1.456 (1.323–1.602) in the univariate Cox regression analysis (*P* < 0.001, Fig. 4A) and 1.412 (1.271–1.568) in the multivariate Cox regression analysis (*P* < 0.001, Fig. 4B), showing that FerRLSig was an independent prognostic indicator. In addition, the predictive accuracy of the model was assessed by a time-dependent receiver operating characteristic ROC analysis at 1, 3, and 5 years, with area under the curve (AUC) values of 0.781, 0.712, and 0.761, respectively (Fig. 4C). Then, we performed a PCA to compare the low- and high-risk groups based on the whole genome, ferroptosis-related lncRNAs, and the risk model. As shown in Fig. 4D and E, the high- and low-risk groups could not be effectively discriminated using the whole genome or ferroptosis-related lncRNAs; however, using FerRLSig, the high- and low-risk patients could be clearly distinguished, further supporting the accuracy of the model. These results indicated that FerRLSig is a significant independent prognostic risk factor for patients with LUAD.

### Correlations between the risk score and clinicopathological factors

**Fig. 5:**
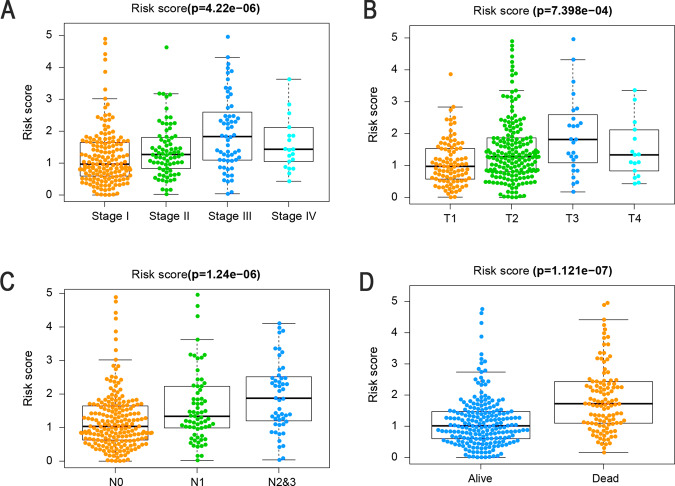
FerRLSig was associated with the clinicopathological features of patients with LUAD. **A** Ferroptosis-related lncRNAs in the cohorts stratified by tumor stage (*P* < 0.001). **B** Ferroptosis-related lncRNAs in the cohorts stratified by tumor size (*P* < 0.001). **C** Ferroptosis-related lncRNAs in the cohorts stratified by regional lymph nodes (*P* < 0.001). **D** Ferroptosis-related lncRNAs in the cohorts stratified by survival outcome (*P* < 0.001). T, tumor size; N, regional lymph node metastasis.

To further assess the roles of FerRLSig in the development of LUAD, we assessed the correlations between the risk score and clinicopathological factors. As shown in Fig. 5 and Table S3, there were significant correlations between the risk score and pathologic stage (*P* < 0.05). In particular, the risk score was significantly higher for stages II–IV than for stage I (Fig. 5A, *P* < 0.001), and the signature was associated with tumor stage (Fig. 5B, *P* < 0.001) and for patients with lymph node metastasis than for those without lymph node metastasis (Fig. 5C, *P* < 0.001). Figure 5D illustrates that patients with a high-risk score (i.e., >1.17) had a significantly poorer prognosis in terms of survival status than patients with low-risk scores (*P* < 0.0001). These results indicate that FerRLSig is closely associated with LUAD progression and prognosis.

### Construction of a predictive nomogram

**Fig. 6:**
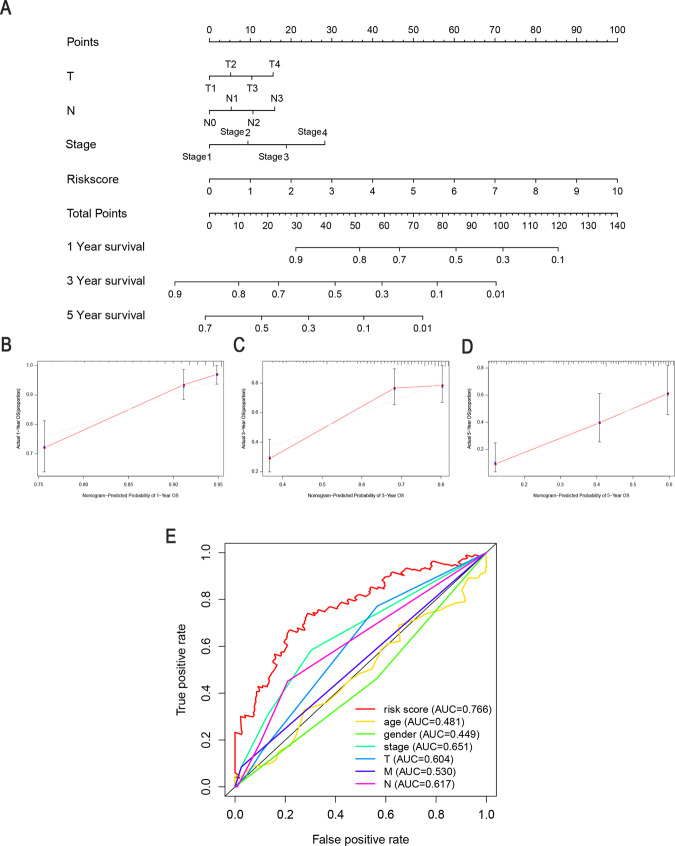
Clinical prognostic nomogram for survival prediction. **A** A clinical prognostic nomogram was developed to predict 1-, 3-, and 5-year survival. Calibration curves showing nomogram predictions for 1-year (**B**), 3-year (**C**), and 5-year (**D**) survival. **E** Time-dependent ROC curve analyses for predicting OS at 5 years by risk score age, sex, stage, T stage (tumor size), M stage (distant metastasis), and N stage (lymph node metastasis).

We constructed a clinically adaptable nomogram to estimate the 1-, 3-, and 5-year survival probabilities of patients with LUAD using FerRLSig combined with other clinicopathological factors (Fig. 6A). The calibration plots of the nomogram for 1-year, 3-years, and 5-years (Fig. 6B–D) indicated that the mortality estimated by the nomogram was close to the actual mortality. Time-dependent ROC curves of 5-year OS were generated, and the AUC value for the clinical prognostic nomogram was 0.766, which was significantly higher than those for age, sex, tumor stage, tumor T stage, tumor M stage, and tumor N stage, further supporting the discriminative ability of FerRLSig in conjunction with clinicopathological factors for predicting survival in LUAD (Fig. 6E).

### Identification of FerRLSig-associated biological pathways

**Fig. 7:**
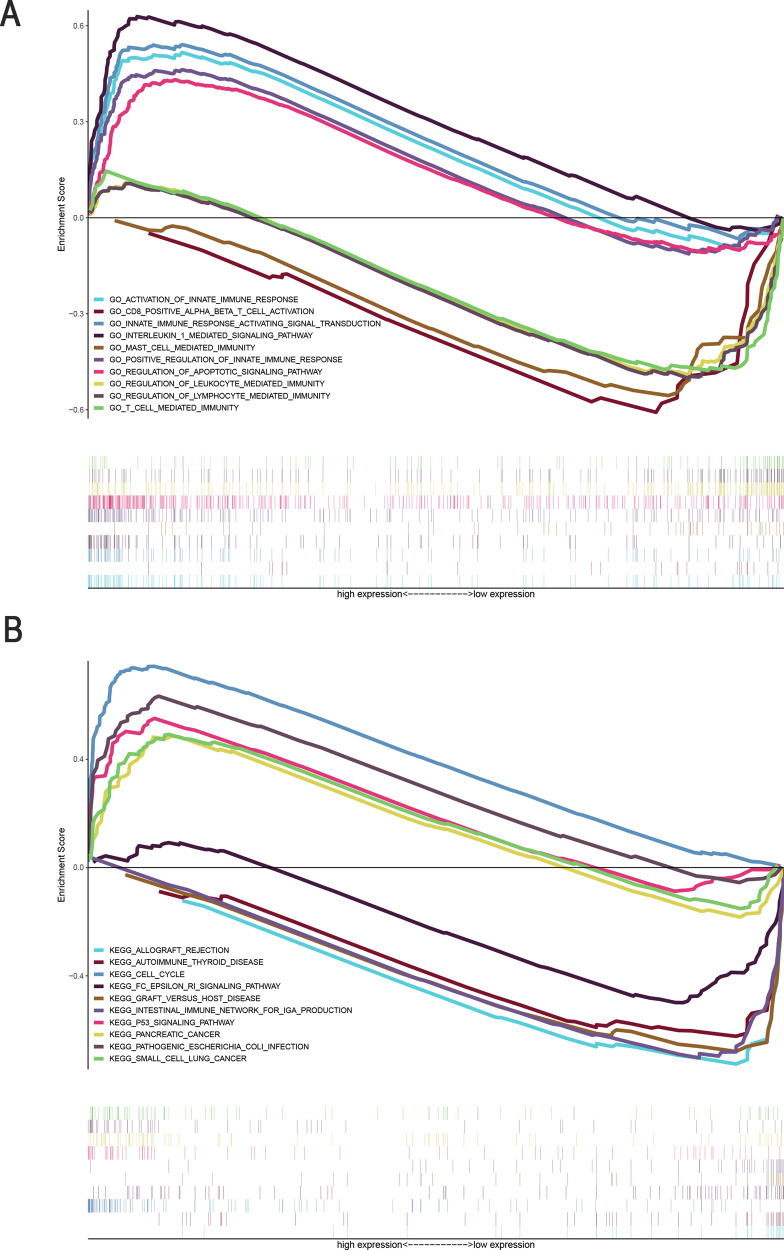
Enrichment plots from gene set enrichment analysis (GSEA). **A** GSEA results showing differential enrichment of genes in GO with ferroptosis-related lncRNAs (5 GO items, namely, positive regulation of activation of innate immune response, innate immune response activating signal transduction, interleukin 1 mediated signaling pathway, positive regulation of innate immune response, and regulation of apoptotic signaling pathway), showed significantly differential enrichment in the high expression phenotype. Five GO items, namely, CD8-positive alpha beta T cell activation, mast cell-mediated immunity, regulation of leukocyte-mediated immunity, regulation of lymphocyte-mediated immunity, and T cell-mediated immunity, showed significantly differential enrichment in the low expression phenotype. **B** GSEA results showing differential enrichment of genes in KEGG with ferroptosis-related lncRNA expression. (5 KEGG items namely, positive regulation of pancreatic cancer, cell cycle, p53 signaling pathway, pathogenic *Escherichia coli* infection, and small cell lung cancer were significantly differential enrichment in high expression phenotype; 5 KEGG items namely, ferroptosis -related lncRNA, FC epsilon RI signaling pathway, autoimmune thyroid disease, and allograft rejection and graft versus host disease showed significantly differential enrichment in the ferroptosis-related lncRNA low-expression phenotype based on the normalized enrichment score (NES), nominal *p* value (NOM *P* value), and FDR value).

Functional annotation was further performed using GSEA. The Gene Ontology (GO) terms activation of innate immune response (NES = 1.95, *P* = 0.004), innate immune response activating signal transduction (NES = 2.00, *P* = 0.000), positive regulation of innate immune response (NES = 1.76, *P* = 0.0014), interleukin 1 mediated signaling pathway (NES = 2.22, *P* = 0.000), and regulation of apoptotic signaling pathway (NES = 1.90, *P* = 0.000) were enriched in LUAD samples with high-risk scores. In contrast, CD8+ alpha beta T cell activation (NES = −1.86, *P* = 0.012), T cell-mediated immunity (NES = −1.74, *P* = 0.033), MAST cell-mediated immunity (NES = −1.88, *P* = 0.012), regulation of leukocyte-mediated immunity (NES = −1.83, *P* = 0.026), and regulation of lymphocyte-mediated immunity (NES = −1.82, *P* = 0.017) were enriched in LUAD samples with low-risk scores (Table S4, Fig. 7A). In addition, 67 enriched Kyoto Encyclopedia of Genes and Genomes (KEGG) pathways were identified. Cell cycle (NES = 2.31, *P* = 0.000), pancreatic cancer (NES = 1.82, *P* = 0.017), p53 signaling pathway (NES = 2.10, *P* = 0.0004), pathogenic *Escherichia coli* (NES = 2.08, *P* = 0.000), and small cell lung cancer (NES = 1.85, *P* = 0.0008) signaling pathways were enriched in the high-risk group. Several immune response pathways, such as the intestinal immune network for IgA production (NES = –1.95, *P* = 0.01), FC epsilon RI signaling pathway (NES = −1.51, *P* = 0.004), autoimmune thyroid disease (NES = −1.87, *P* = 0.008), allograft rejection (NES = −1.81, *P* = 0.016), and graft versus host disease (NES = −1.74, *P* = 0.027), were enriched in the low-risk group (Table S5, Fig. 7B). These results indicate that the lncRNAs in the newly developed signature may be associated with the tumor immune microenvironment.

### Differences in the response to immunotherapy, chemotherapy, and targeted therapy between high-risk and low-risk groups

**Fig. 8:**
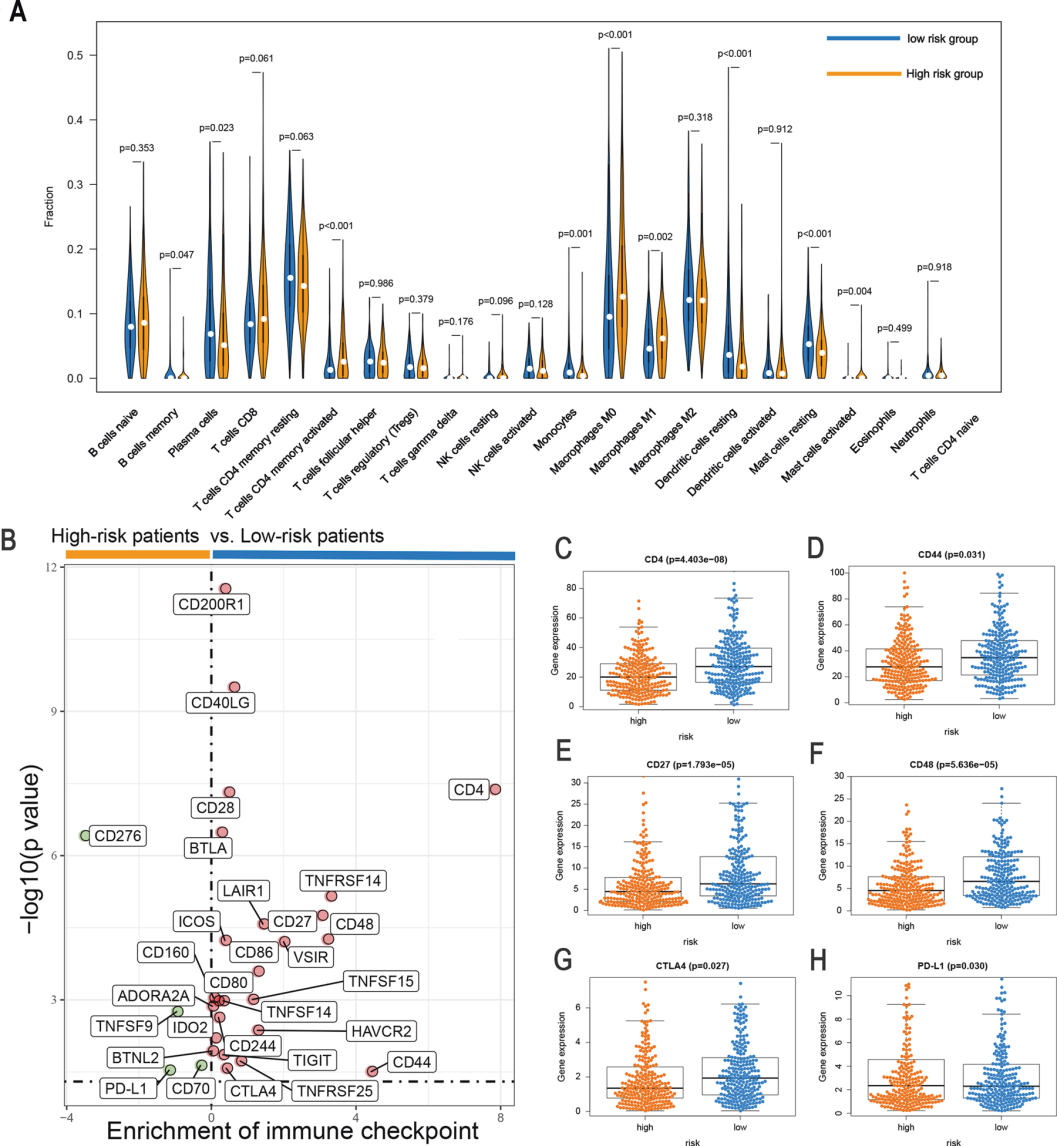
Comparison of the immune microenvironment and immune checkpoints with LUAD between the high-risk and low-risk groups. **A** Violin plot of immune-infiltrating lymphocytes between the low-risk and high-risk groups, in which orange indicates high-risk samples and blue indicates low-risk samples. **B** Volcano plots for the enrichment of expression of immune checkpoints between the low-risk and high-risk groups. The differential expression of six immune checkpoints, (**C**) CD4, (**D**) CD44, (**E**) CD27, (**F**) CD48, (**G**) CTLA4, and (**H**) PD-L1, between the high-risk group and the low-risk group. Orange indicates high-risk patients, and blue indicates low-risk patients.

To assess the correlations between the risk score and tumor immune cell infiltration, the CIBERSORT algorithm was applied to compare the proportions of 22 immune cell types between the high-risk and low-risk groups. Differences in the infiltration of 22 immune cell types among patients with LUAD from TCGA are shown in Fig. S1A, showing that this is an intrinsic feature reflecting individual differences. The proportions of immune cells differed between the two risk groups (Fig. S1B, where colors ranging from green to red represent the infiltration density from low to high). Patients with LUAD in the high-risk group had higher ratios of activated memory CD4 T cells (*P* < 0.001), M0 macrophages (*P* < 0.001), M1 macrophages (*P* = 0.002), and activated mast cells (*P* = 0.005). In contrast, memory B cells (*P* = 0.047), plasma cells (*P* = 0.023), monocytes (*P* = 0.001), resting dendritic cells (*P* < 0.001), resting mast cells (*P* < 0.001), and monocytes (*P* = 0.002) were negatively correlated with the risk score (Fig. 8A). The substantial immune infiltration observed in the low-risk group partly reflected the reduction in malignancy and effects of various treatments, indicating that our signature is not only a prognostic marker but also reflects levels of immune cell infiltration.

The expression levels of immune checkpoints and/or their ligands may constitute predictive biomarkers for immune checkpoint blockade therapy. We further investigated the relationship between the levels of 29 immune checkpoint inhibitor (ICI) genes and the two risk groups stratified by FerRLSig. Low-risk patients tended to express higher levels of 25 immune checkpoint genes, including *ADORA2A, BTLA, CD160, CD200R1, CD244, CD28, CD40LG, CD70, CD80, CD86, HAVCR2, ICOS, IDO2, TIGIT, TNFRSF14, TNFRSF25, TNFSF14, TNFSF15*, and *VSIR*, while the other four immune checkpoint genes (*CD276, TNFSF9, PD-L1*, and *CD70*) were highly expressed in high-risk patients (Fig. 8B and Fig. S2, *P* < 0.05). These results indicate that FerRLSig is a candidate biomarker for ICI therapy.

In addition, we evaluated the associations between the risk score and the efficacy of targeted therapeutics and chemotherapeutics for LUAD. Patients with low-risk scores were highly sensitive to the targeted therapeutics gefitinib (*P* = 0.008) and erlotinib (*P* < 0.001). In addition, patients with low-risk scores were highly sensitive to the chemotherapeutics cisplatin (*P* = 0.0068), etoposide (*P* < 0.001), paclitaxel (*P* < 0.001), docetaxel (*P* < 0.001), and gemcitabine (*P* = 0.001), suggesting that FerRLSig is a potential predictor of chemosensitivity (Fig. S3). These results could also explain the poor prognosis of patients with high risk scores, as these patients are likely to be more resistant to chemotherapy and targeted therapy.

## Discussion

Lung cancer is the leading cause of cancer-related mortality worldwide. The pathogenesis of LUAD is unclear, contributing to the lack of effective treatments [24, 25]. Compared with single clinical biomarkers, the integration of multiple biomarkers into a single model can improve predictive accuracy and assist in the development of individualized treatment plans.

Ferroptosis is a novel iron-dependent and nonapoptotic form of cell death that is mediated by oxidative modifications of membrane polyunsaturated phospholipids. As an adaptive mechanism to eliminate malignant cells, ferroptosis is a promising new target for cancer treatment [26, 27]. Battaglia et al. [28] reported that ferroptosis plays an important role in cancer. Various lncRNAs and microRNAs (miRNAs) involved in the regulation of ferroptosis have been identified [18, 29]. For example, the lncRNA P53RRA binds to Ras GTPase-activating protein-binding protein 1 (G3BP1) and prevents its interaction with p53 to induce cell cycle arrest, apoptosis, and ferroptosis in lung cancer [20].

Although several lncRNA signatures associated with lung cancer have been reported, previous studies have focused on relapse-free survival or OS for patients with early-stage lung cancer [30, 31], and general prognostic models for lung cancer remain warranted. In the present study, we constructed a prognostic model using 10 ferroptosis-related lncRNAs (AL606489.1, AC106047.1, LINC02081, AC090559.1, AC026355.1, FAM83A-AS1, AL034397.3, AC092171.5, AC010980.2, and AC123595.1). Based on the ROC curve, the lncRNA signature had a moderate predictive performance for OS. In addition, our newly developed nomogram is expected to improve clinical decision-making and guide the development of treatment strategies.

Among the identified lncRNAs, five were associated with tumor progression, namely, AC026355.1, FAM83A-AS1, AL034397.3, AC092171.5, and AC123595.1. FAM83A-AS1 promotes the migration and invasion of tumor cells in LUAD [32, 33]. AL034397.3, AC026355.1, and AC123595.1 are components of a lncRNA risk model related to immunity, suggesting a possible link between ferroptosis and immune regulation in cancer [34, 35]. However, studies of the prognostic value in cancer and contributions to ferroptosis are lacking for five lncRNAs (AL606489.1, AC106047.1, LINC02081, AC090559.1, and AC010980.2). Thus, additional studies are needed to explore the effects of these lncRNAs in LUAD and ferroptosis.

The most significant contribution of this research is the demonstration of the relationship between FerRLSig and the tumor immune microenvironment. Notably, the complex interplay between tumor cells and the tumor microenvironment not only plays a pivotal role in tumor development but also has significant effects on immunotherapeutic efficacy and overall survival [36, 37]. In this study, a functional enrichment analysis indicated that ferroptosis-related lncRNAs are primarily involved in immune pathways. Patients with high-risk scores had higher proportions of activated memory CD4 T cells, M0 macrophages, M1 macrophages, and mast cells, confirming the roles of ferroptosis-related lncRNAs in the regulation of tumor immune infiltration. Since our results link FerRLSig to immune infiltration in LUAD, these ferroptosis-related lncRNAs may be targets for combined treatments with immune checkpoint inhibitors.

The combination of immune checkpoint blockade with immunotherapies such as CTLA-4, PD-1, and PD-L1 inhibitors is a promising approach to treat a variety of malignancies, and an activated tumor immune microenvironment is correlated with good outcomes of immune checkpoint inhibitor treatment [38, 39]. Interestingly, PD-L1 was highly expressed in the high-risk group, indicating that patients with high-risk scores may benefit more from anti-PD-L1 immunotherapy, while CTLA-4 was highly expressed in the low-risk group, indicating that patients in the low-risk group may benefit more from anti-CTLA-4 immunotherapy. This provides new insights into tumor immunotherapy.

The current study had several limitations. First, we used a single data source. Second, it was a retrospective study. Third, well-known prognostic factors such as chemotherapy data and tumor markers were not included in the nomogram since data for these parameters were incomplete. Thus, additional prospective studies are needed to verify the prognostic value of FerRLSig. In addition, functional experiments should be conducted to further elucidate the molecular mechanisms underlying the effects of ferroptosis-related lncRNAs.

In summary, we constructed a ferroptosis-related lncRNA signature for LUAD to predict prognosis. We established an effective nomogram including FerRLSig. Importantly, the FerRLSig generated in our study might be associated with immune infiltration levels and even the efficacy of tumor immunotherapy.

## Materials and methods

### Datasets and sample extraction

RNA-sequencing (RNA-seq) data for LUAD were obtained from The Cancer Genome Atlas (TCGA) database (https://portal.gdc.cancer.gov/). A total of 535 patients with LUAD were included in the study. In addition, complete clinical information was downloaded from TCGA. A total of 477 patients with complete follow-up information and survival time >30 days and 325 patients with complete clinicopathological data were included in subsequent analyses.

### Ferroptosis-related gene detection

In total, 174 ferroptosis-related genes were collected from the FerrDb database (http://www.zhounan.org/ferrdb) and a literature search [7, 14, 26, 40–42]. Ultimately, 169 ferroptosis-related genes were retrieved on the basis of available mRNA expression data for LUAD in TCGA (Table S1).

### Bioinformatics analysis

Coexpression networks were visualized using Cytoscape 3.7.2, while principal component analysis (PCA) was conducted to evaluate the distribution of patients with different risk scores. The R package scatterplot3D was used to generate the PCA plots. The nomogram was developed and validated using the rms package in R version 4.0.3 (http://www.r-project.org/); the R package ggalluvial was used to obtain the Sankey map.

### Identification of a prognostic ferroptosis-related lncRNA model for LUAD

A total of 1621 ferroptosis-related lncRNAs were identified by constructing a ferroptosis-related mRNA–lncRNA coexpression network using |Pearson correlation coefficient | > 0.3 and *P* < 0.001 as thresholds. The coexpression networks were visualized using Cytoscape 3.7.2. Then, based on univariate analyses with *P* < 0.01 as the threshold, significant prognostic ferroptosis-related lncRNAs were identified and subsequently incorporated into a multivariate Cox regression analysis to establish risk scores. The risk score for each patient was calculated using the following formula: risk score = ∑i coefficient(lncRNA1) × expression(lncRNA1) + coefficient(lncRNA2) × expression(lncRNA2) + …… + coefficient(lncRNAn) × expression(lncRNAn). A linear regression analysis was used to evaluate the relationship between survival and high-risk and low-risk groups. The consistency index (c-index), calibration curve, and receiver operating characteristic (ROC) curves were used to explore the accuracy of the model. Demographic data were included in multivariate Cox regression analysis to confirm whether the risk score is an independent prognostic indicator. The distribution of survival statuses according to risk score levels was evaluated.

### Statistical analysis

All computational and statistical analyses were conducted using R (version 4.0.3). Survival analysis was performed using the Kaplan–Meier method. The Kruskal–Wallis test was used to compare differences between groups. The chi-squared test or Fisher’s exact test was used for analyses of clinical information. Spearman or Pearson correlation coefficients were used to evaluate the relationships among lncRNA expression, immune infiltration, and immune checkpoint gene expression.

### Significance of the model for the prediction of clinical treatment response

Levels of immune cell infiltration were quantified in each LUAD sample using CIBERSORT (http://cibersort.stanford.edu/). The relationships between the FerRLSig score and the expression levels of ICI genes were determined using the ggstatsplot package in R, and violin plots were generated for visualization. IC_50_ values for various antitumor drugs recommended for lung cancer treatment, such as cisplatin, etoposide, docetaxel, gefitinib, erlotinib, gemcitabine, and paclitaxel, were compared between groups using the Wilcoxon signed-rank test using pRRophetic and ggplot2 in R.

### Gene set enrichment analysis

Gene set enrichment analysis (GSEA) was performed for the high-risk and low-risk groups according to the prognostic model. Significant predefined biological processes and pathways were enriched when NOM *P* < 0.05 and false discovery rate (FDR) < 0.25. c5.go. v7.1 symbols.gmt and c2.cp.kegg.v7.1.symbols.gmt were selected from the Molecular Signatures Database (MSigDB, http://software.broadinstitute.org/gsea/msigdb/index.jsp) as the reference files.

## Supplementary information

Supplementary Table S1

Supplementary Table S2

Supplementary Table S3

Supplementary Table S4

Supplementary Table S5

Supplementary Figures and Tables.

Supplementary Fig. S1

Supplementary Fig. S2

Supplementary Fig. S3

## Data Availability

The datasets generated and analyzed during the current study are available in the Figshare (https://figshare.com/s/f34a12c9a77534612ea7) repository.
