# Supplementary material for: Identification of a prognostic ferroptosis-related lncRNA signature in the tumor microenvironment of lung adenocarcinoma
Source: Cell Death Discov. 2021 Jul 26;7:190. doi: 10.1038/s41420-021-00576-z (PMC8313561; doi:10.1038/s41420-021-00576-z)
Supplement: Supplementary file 1 — Supplementary Table S1 [file 41420_2021_576_MOESM1_ESM.docx]

**Table S1** A list of ferroptosis -related genes.

| AGPAT3 |
| --- |
| ALB |
| ALOX12 |
| ALOX15 |
| ALOX5 |
| ANGPTL7 |
| ARRDC3 |
| ASNS |
| ATF3 |
| ATF4 |
| ATP5MC3 |
| ATP6V1G2 |
| AURKA |
| BLOC1S5-TXNDC5 |
| BNIP3 |
| CAPG |
| CBS |
| CEBPG |
| CHAC1 |
| DDIT3 |
| DDIT4 |
| DRD4 |
| DRD5 |
| DUSP1 |
| EIF2AK4 |
| EIF2S1 |
| ELAVL1 |
| FTH1 |
| FTL |
| GABPB1 |
| GDF15 |
| GPT2 |
| GPX2 |
| GPX4 |
| HBA1 |
| HERPUD1 |
| HIC1 |
| HMOX1 |
| HNF4A |
| HSD17B11 |
| JDP2 |
| KLHL24 |
| LURAP1L |
| MAP3K5 |
| MAPK14 |
| MT3 |
| NCF2 |
| NGB |
| NNMT |
| NOS2 |
| OXSR1 |
| PCK2 |
| PLIN4 |
| PRDX1 |
| PSAT1 |
| PTGS2 |
| RGS4 |
| RIPK1 |
| RPL8 |
| RRM2 |
| SELENOS |
| SESN2 |
| SETD1B |
| SLC1A4 |
| SLC2A1 |
| SLC2A12 |
| SLC2A14 |
| SLC2A3 |
| SLC2A6 |
| SLC2A8 |
| SLC3A2 |
| SLC7A11 |
| SLC7A5 |
| SRXN1 |
| STMN1 |
| TF |
| TFRC |
| TRIB3 |
| TSC22D3 |
| TUBE1 |
| TXNIP |
| TXNRD1 |
| UBC |
| VEGFA |
| VLDLR |
| XBP1 |
| YWHAE |
| ZFP69B |
| ZNF419 |
| HMGB1 |
| MAFG |
| ACSL4 |
| AKR1C1 |
| AKR1C2 |
| AKR1C3 |
| CARS |
| CD44 |
| CISD1 |
| CS |
| DPP4 |
| FANCD2 |
| GCLC |
| GCLM |
| GLS2 |
| GSS |
| HMGCR |
| HSPB1 |
| CRYAB |
| LPCAT3 |
| MT1G |
| NCOA4 |
| SAT1 |
| FDFT1 |
| TP53 |
| EMC2 |
| AIFM2 |
| PHKG2 |
| HSBP1 |
| ACO1 |
| STEAP3 |
| NFS1 |
| ACSL3 |
| ACACA |
| PEBP1 |
| ZEB1 |
| SQLE |
| FADS2 |
| NFE2L2 |
| KEAP1 |
| NQO1 |
| NOX1 |
| ABCC1 |
| SLC1A5 |
| GOT1 |
| G6PD |
| PGD |
| IREB2 |
| ACSF2 |
| CCBR1 |
| Nrf2 |
| LSH |
| TFR1 |
| ACSL1 |
| ACSL5 |
| ACSL6 |
| ATG5 |
| ATG7 |
| CDKN1A |
| CP |
| CUL3 |
| CYBB |
| FTMT |
| MAP1LC3A |
| MAP1LC3B |
| MAP1LC3C |
| PCBP1 |
| PCBP2 |
| PRNP |
| SAT2 |
| SLC11A2 |
| SLC39A14 |
| SLC39A8 |
| SLC40A1 |
| VDAC2 |
| VDAC3 |
| EGLN1 |
| BECN1 |
| BAP1 |
| HSF1 |
| HIF1A |
| ROS1 |
| ARF6 |
| SCD |
