# Supplementary material for: Identification of a prognostic ferroptosis-related lncRNA signature in the tumor microenvironment of lung adenocarcinoma
Source: Cell Death Discov. 2021 Jul 26;7:190. doi: 10.1038/s41420-021-00576-z (PMC8313561; doi:10.1038/s41420-021-00576-z)
Supplement: Supplementary file 2 — Supplementary Table S2 [file 41420_2021_576_MOESM2_ESM.docx]

| Gene ID | KM | B | SE | HR | HR.95L | HR.95H | *P*-value |
| --- | --- | --- | --- | --- | --- | --- | --- |
| AL606489.1 | 0.002222 | 0.261048 | 0.059865 | 1.29829 | 1.154554 | 1.45992 | 1.30E-05 |
| CRNDE | 0.001338 | -0.04641 | 0.015487 | 0.954647 | 0.926105 | 0.98407 | 0.002728 |
| AC106047.1 | 0.009234 | -0.39591 | 0.141703 | 0.67307 | 0.50985 | 0.888541 | 0.005207 |
| LINC02081 | 0.007875 | 0.113283 | 0.041562 | 1.119948 | 1.032334 | 1.214999 | 0.006418 |
| AP000695.2 | 0.00327 | 0.339448 | 0.092384 | 1.404172 | 1.17161 | 1.682898 | 0.000238 |
| AC090559.1 | 0.001997 | -0.21642 | 0.079552 | 0.805398 | 0.689122 | 0.941294 | 0.006519 |
| LINC01843 | 0.005575 | 0.104427 | 0.028139 | 1.110075 | 1.05051 | 1.173017 | 0.000206 |
| AL691432.2 | 0.000687 | -0.17918 | 0.052852 | 0.835954 | 0.753693 | 0.927192 | 0.000698 |
| AC026355.1 | 0.000146 | -0.27281 | 0.081788 | 0.761238 | 0.648489 | 0.893591 | 0.000851 |
| FAM83A-AS1 | 0.009635 | 0.040105 | 0.011009 | 1.04092 | 1.018701 | 1.063624 | 0.000269 |
| AP000695.1 | 0.007595 | 0.226476 | 0.066422 | 1.254172 | 1.101079 | 1.42855 | 0.00065 |
| AL034397.3 | 0.000174 | -0.30254 | 0.109236 | 0.738936 | 0.596519 | 0.915353 | 0.005612 |
| AC087752.3 | 0.004821 | -0.43634 | 0.148751 | 0.646397 | 0.482927 | 0.8652 | 0.003353 |
| VIM-AS1 | 0.001771 | -0.18652 | 0.063224 | 0.829846 | 0.733131 | 0.93932 | 0.003177 |
| HLA-DQB1-AS1 | 0.002643 | -0.07749 | 0.022276 | 0.925439 | 0.885903 | 0.96674 | 0.000504 |
| AC092171.5 | 0.002081 | -0.20708 | 0.071638 | 0.812957 | 0.706462 | 0.935505 | 0.003845 |
| AC010980.2 | 0.00339 | 0.287954 | 0.086634 | 1.333696 | 1.125418 | 1.580519 | 0.000888 |
| LINC00996 | 0.007527 | -0.53738 | 0.204222 | 0.584277 | 0.391548 | 0.871873 | 0.008505 |
| AC123595.1 | 0.003583 | -0.84537 | 0.25053 | 0.429398 | 0.26279 | 0.701635 | 0.00074 |
| AC011477.2 | 3.74E-05 | -0.25857 | 0.074085 | 0.772157 | 0.667798 | 0.892826 | 0.000483 |
| HSPC324 | 0.009714 | -0.61132 | 0.203973 | 0.542636 | 0.36382 | 0.809341 | 0.002726 |

**Supplementary Table S2** Univariate cox results of ferroptosis-related lncRNAs in LUAD.
