# Supplementary material for: Identification of a prognostic ferroptosis-related lncRNA signature in the tumor microenvironment of lung adenocarcinoma
Source: Cell Death Discov. 2021 Jul 26;7:190. doi: 10.1038/s41420-021-00576-z (PMC8313561; doi:10.1038/s41420-021-00576-z)
Supplement: Supplementary file 3 — Supplementary Table S3 [file 41420_2021_576_MOESM3_ESM.docx]

**Supplementary Table S3** Correlation between the risk score and clinicopathological factors in LUAD.

| Clinical | Group | n | Risk score | | t | *P* |
| --- | --- | --- | --- | --- | --- | --- |
|  |  |  | Mean | SD |  |  |
| age | > 65 | 168 | 1.476 | 1.127 | -0.30573 | 0.76 |
|  | ≤65 | 157 | 1.519 | 1.371 |  |  |
| gender | Female | 164 | 1.548 | 1.338 | 0.746797 | 0.456 |
|  | Male | 161 | 1.444 | 1.153 |  |  |
| stage | I-II | 245 | 1.32 | 1.116 | -3.99852 | <0.001 |
|  | III-IV | 80 | 2.037 | 1.471 |  |  |
| T | T1-2 | 281 | 1.423 | 1.181 | -2.20687 | 0.032 |
|  | T3-4 | 44 | 1.964 | 1.557 |  |  |
| M | M0 | 304 | 1.461 | 1.206 | -1.45926 | 0.159 |
|  | M1 | 21 | 2.017 | 1.717 |  |  |
| N | N0 | 205 | 1.26 | 1.044 | -4.22749 | <0.001 |
|  | N1 | 120 | 1.901 | 1.456 |  |  |
