# Supplementary material for: Identification of a prognostic ferroptosis-related lncRNA signature in the tumor microenvironment of lung adenocarcinoma
Source: Cell Death Discov. 2021 Jul 26;7:190. doi: 10.1038/s41420-021-00576-z (PMC8313561; doi:10.1038/s41420-021-00576-z)
Supplement: Supplementary file 6 — Supplementary Figures and Tables. [file 41420_2021_576_MOESM6_ESM.doc]

**Supplementary Figure S1. Estimation of immune cell fractions using the CIBERSORT algorithm.** (**A**) Twenty-one immune cells were annotated by various colors indicated in the legend. (**B**) Correlations between tumor-infiltrating immune cells. Red represents a positive correlation and blue represents a negative correlation.

**Supplementary Figure S2. Comparison of expression levels of 23 immune checkpoint genes between patients with LUAD in the high-risk group and low-risk group.**

The expression levels of some immune checkpoint genes, namely (**A**) CD28 (*P* < 0.001) and (**C**) BTLA (*P* < 0.001), (**D**) BTNL2 (*P* < 0.01), (**E**) ADORA2A (*P* < 0.001) (**F**) CD40LG (*P* < 0.001), (**G**) TNFRSF14 (*P* < 0.001), (**H**) TNFRSF25 (*P* < 0.05), (**I**) TNFSF14 (*P* < 0.01), (**J**) VSIR (*P* < 0.001), (**K**) CD244 (*P* < 0.01), (**L**) TIGIT (*P* < 0.05), (**M**) TNFSF15 (*P* < 0.001), (**N**) CD80 (*P* < 0.01), (**O**) LAIR1 (*P* < 0.001), (**P**) IDO2 (*P* < 0.01), (**Q**) ICOS(*P* < 0.001), (**R**) HAVCR2 (*P* < 0.01), (**S**) CD200R1 (*P* < 0.001), (**T**) CD160 (*P* < 0.001), and (**U**) CD86 (*P* < 0.001) were significantly higher in low-risk samples than that in low-risk samples. Conversely, the expression of immune checkpoints (**B**) CD70 (*P* < 0.05), (**S**) CD276 (*P* < 0.001), and (**W**) TNFSF9 (*P* < 0.01) was significantly higher in the low-risk samples than in the low-risk samples. The expression levels of other immune checkpoint genes, including (**B**) CD70 (*P* < 0.05), (**S**) CD276 (*P* < 0.001), and (**W**) TNFSF9 (*P* < 0.01), were significantly higher in the high-risk samples than in the low-risk samples. The expression levels in the high-risk samples are indicated in orange while in low-risk samples are in blue.

**Supplementary Figure S3.** **Comparison of responses to chemotherapy and targeted therapy between the high‑risk and low‑risk groups**

The model acted as a potential predictor for chemosensitivity, since high risk scores were related to lower IC_50_ values for chemotherapeutics and target-directed treatments, such as (**A**) docetaxel (*P* < 0.001), (**B**) cisplatin (*P* < 0.01), (**C**) paclitaxel (*P* < 0.001), (**D**) gemcitabine (*P* < 0.01), (**E**) gefitinib (*P* < 0.01), (**F**) etoposide (*P* < 0.001), and (**G**) erlotinib (*P* < 0.001).

**Supplementary Table S1.** List of ferroptosis-related genes.

**Supplementary Table S2.** Univariate Cox results for ferroptosis-related lncRNAs in LUAD.

**Supplementary Table S3.** Correlation between the risk score and clinicopathological factors in LUAD.

**Supplementary Table S4.** GO pathway analysis results according to the signature.

**Supplementary Table S5.** KEGG pathway analysis results based on the signature.
