# Supplementary figures and images for: Identification of a prognostic ferroptosis-related lncRNA signature in the tumor microenvironment of lung adenocarcinoma
Source: Cell Death Discov. 2021 Jul 26;7:190. doi: 10.1038/s41420-021-00576-z (PMC8313561; doi:10.1038/s41420-021-00576-z)

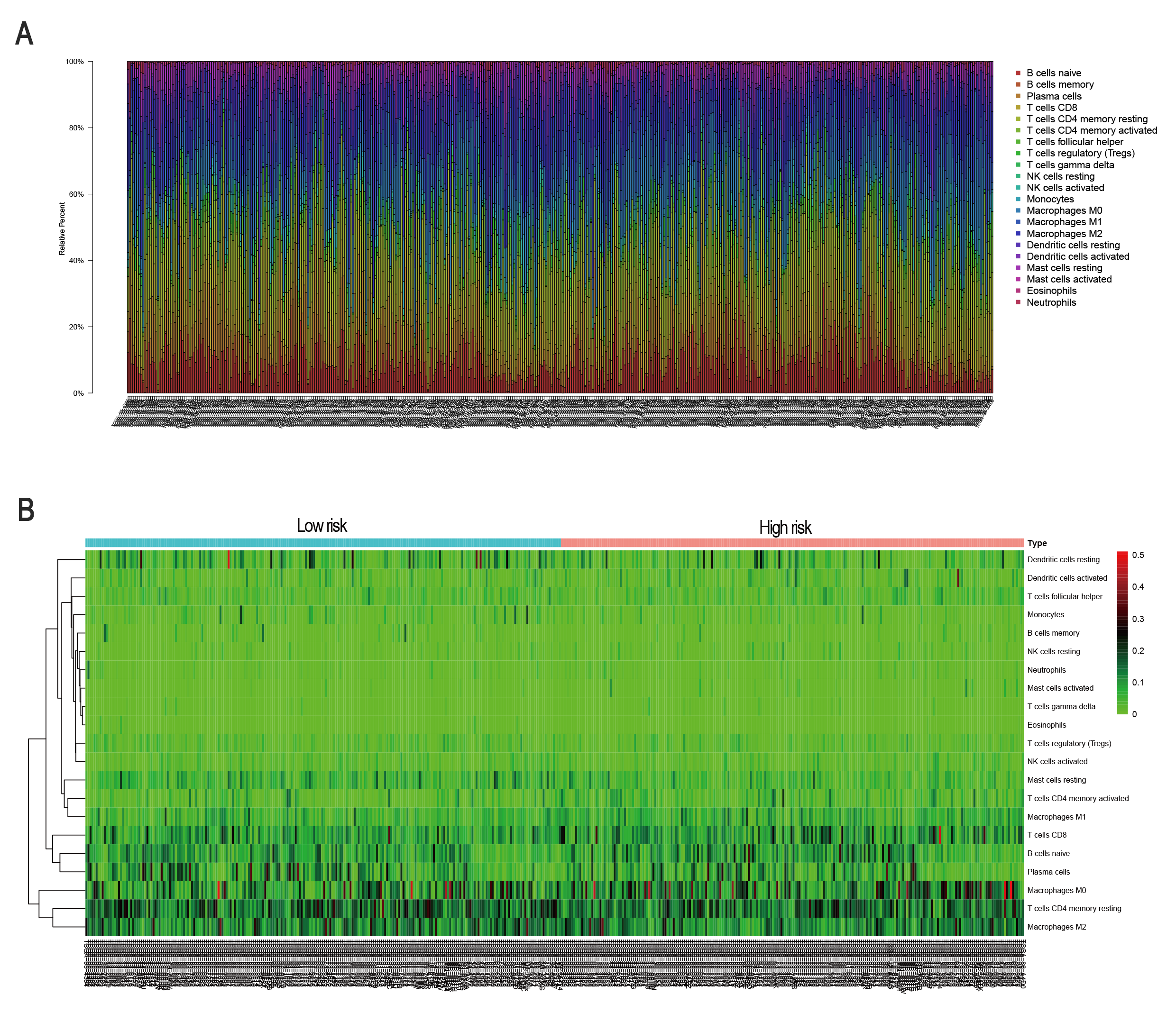

Supplement: Supplementary file 7 — Supplementary Fig. S1 [file 41420_2021_576_MOESM7_ESM.tif]

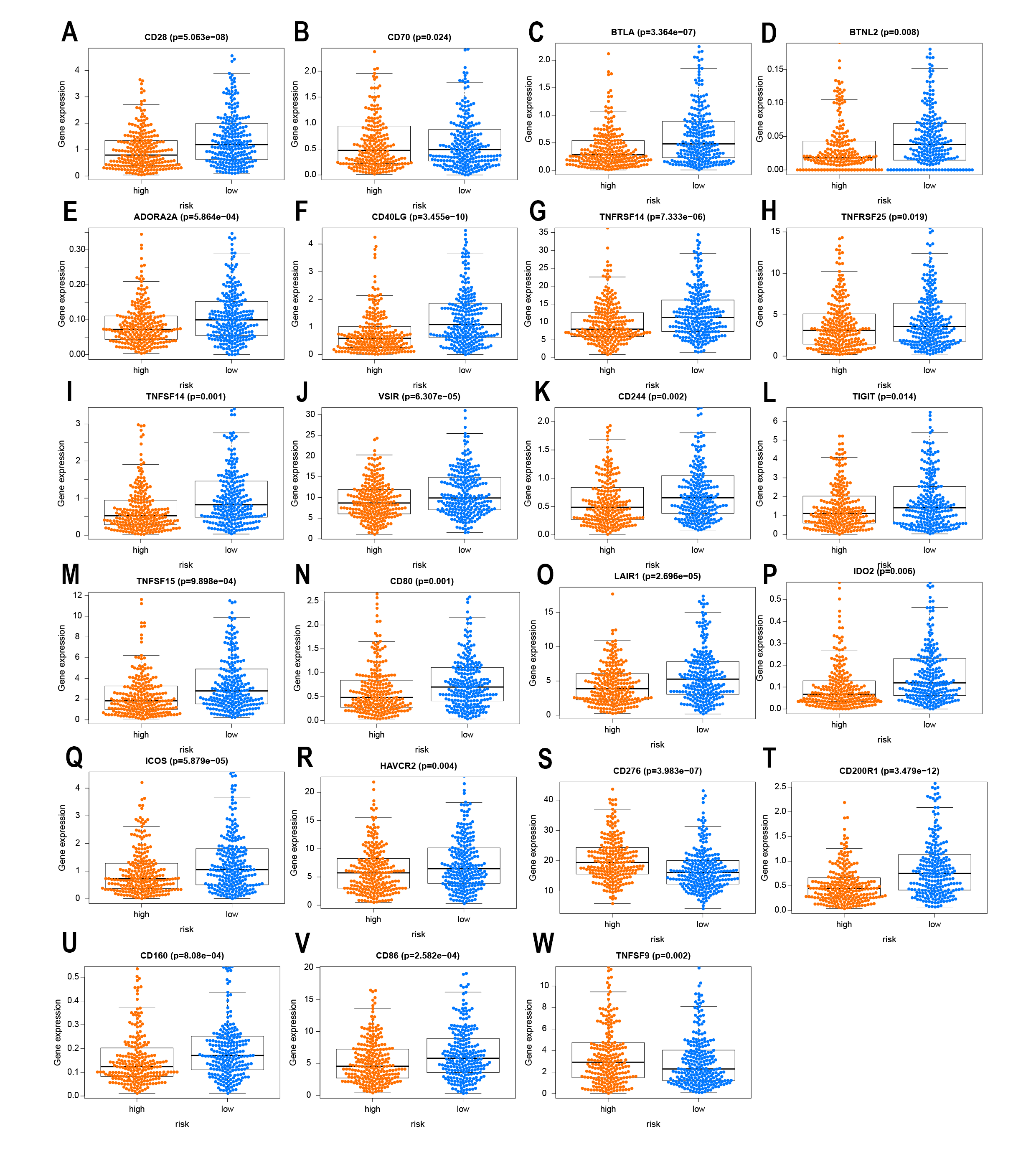

Supplement: Supplementary file 8 — Supplementary Fig. S2 [file 41420_2021_576_MOESM8_ESM.tif]

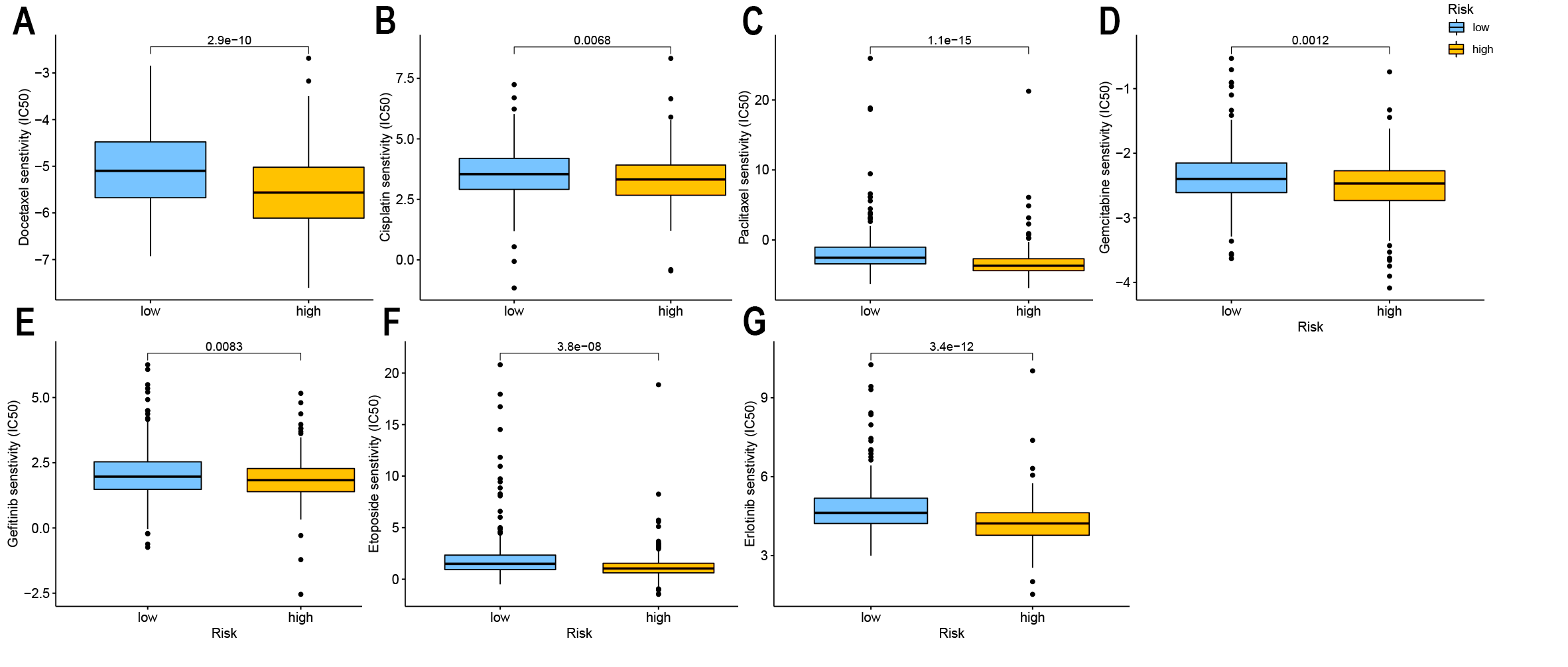

Supplement: Supplementary file 9 — Supplementary Fig. S3 [file 41420_2021_576_MOESM9_ESM.tif]
